# Cross-species Analysis Reveals Evolving and Conserved Features of the Nuclear Factor κB (NF-κB) Proteins* [Fn FN2]

**DOI:** 10.1074/jbc.M113.451153

**Published:** 2013-03-18

**Authors:** Grigory Ryzhakov, Ana Teixeira, David Saliba, Katrina Blazek, Tatsushi Muta, Jiannis Ragoussis, Irina A. Udalova

**Affiliations:** From the ‡ Kennedy Institute of Rheumatology, Nuffield Department of Orthopaedics, Rheumatology and Musculoskeletal Sciences, University of Oxford, London W6 8LH, United Kingdom,; the § Wellcome Trust Centre for Human Genetics, University of Oxford, Oxford OX3 7BN, United Kingdom, and; the ¶ Laboratory of Cell Recognition and Response, Department of Developmental Biology and Neurosciences, Graduate School of Life Sciences, Tohoku University, Sendai 980-8577, Japan

**Keywords:** Cell Signaling, Evolution, Protein/Nucleic Acid Interaction, Protein/Protein Interactions, Transcription, IκB, NF-κB, *Nematostella vectensis*, Immune

## Abstract

NF-κB is a key regulator of immune gene expression in metazoans. It is currently unclear what changes occurred in NF-κB during animal evolution and what features remained conserved. To address this question, we compared the biochemical and functional properties of NF-κB proteins derived from human and the starlet sea anemone (*Nematostella vectensis*) in 1) a high-throughput assay of *in vitro* preferences for DNA sequences, 2) ChIP analysis of *in vivo* recruitment to the promoters of target genes, 3) a LUMIER-assisted examination of interactions with cofactors, and 4) a transactivation assay. We observed a remarkable evolutionary conservation of the DNA binding preferences of the animal NF-κB orthologs. We also show that NF-κB dimerization properties, nuclear localization signals, and binding to cytosolic IκBs are conserved. Surprisingly, the Bcl3-type nuclear IκB proteins functionally pair up only with NF-κB derived from their own species. The basis of the differential NF-κB recognition by IκB subfamilies is discussed.

## Teaser

**Background:** NF-κB regulates transcription via binding to DNA and interactions with cofactors.

**Results:** NF-κB binding to DNA and cytosolic IκBs is conserved, whereas binding to nuclear IκBs has evolved.

**Conclusion:** There is distinct evolutionary pressure on two NF-κB/IκB binding interfaces.

**Significance:** The results provide inroads into IκB-specific modulation of NF-κB activity.

## Introduction

Mammalian immunity is a constantly evolving system with multiple levels of redundancy and compensatory mechanisms allowing flexible but robust protection against pathogens. Recent advances in genomics and bioinformatics allow for systematic comparisons of the defense mechanisms in a wide selection of animal taxa. This is critical for an in-depth understanding of the molecular machinery underlying the host defenses against pathogens.

Cnidaria is a diverse phylum of basal animals, including sea anemones, jellyfish, and corals, the important builder species of marine ecosystems (1). As any other animals, cnidarians are attacked by various pathogens (2). It has been shown that a freshwater cnidarian *Hydra* produces antimicrobial peptides in response to pathogen-associated molecular patterns, which serve as an indicator of innate immunity (3).

The starlet sea anemone (*Nematostella vectensis*) is an emerging model system to study physiological processes in basal animals (4). Its recently published genome analysis revealed a remarkably large number of shared genes and genomic linkage groups between *Nematostella* and vertebrates (5). A number of homologs of key mammalian immune molecules were identified in *Nematostella* (6). Many of them have no paralogs present in the genome, which suggests their non-redundant function (7). For example, the *N. vectensis* (Nv)^4^ genome contains just one NF-κB gene, NvNF-κB p50, with p50 and p52 being the closest human homologs (8).

NF-κB is the key regulator of gene expression in immune processes (9, 10). There are five NF-κB subunits in mammals (NF-κB1, NF-κB2, RelA, cRel, and RelB), which regulate gene expression acting as homo- or heterodimers (10). The N-terminal part of NF-κB molecules consists of a Rel homology domain (RHD), which binds to DNA, and a dimerization domain (IPT (Ig-like, plexins, transcription factors)). The C-terminal part of NF-κB forms a transactivation domain in RelA, RelB, and cRel or, alternatively, is represented by an ankyrin repeat domain (ARD) in NF-κB1 p105 and NF-κB2 p100 (11). The latter proteins are also present in proteolytically processed forms, NF-κB1 p50 and NF-κB2 p52 (9, 12). The processing requires a glycine-rich region, which is located between the N-terminal part and the ARD (13).

NF-κB proteins are regulated by a family of cofactors called IκBs (inhibitor of κB proteins) (14). In mammals, NF-κB is sequestered in the cytoplasm as an inactive complex bound to IκB (15). There are three mammalian cytoplasmic IκBs, IκBα, IκBβ, and IκBϵ (14). Upon stimulation, IκBs are degraded, and NF-κB translocates to the cell nucleus to activate gene expression (9, 15). There are also three mammalian nuclear IκBs, Bcl3, IκBζ, and IκBδ, which are involved in the regulation of late NF-κB-dependent genes (16). All IκB proteins are structurally related to the ARDs of NF-κB1/2 (14). Bcl3 and IκBζ have an N-terminal transactivation domain, enabling these proteins, in addition to their inhibitory roles, to function as NF-κB transcription coactivators for certain genes (17, 18). Two IκB genes were found in *Nematostella*, NvIκB and NvBcl3, related to human cytoplasmic IκBϵ and nuclear Bcl3, respectively (19).

Previous reports showed NvNF-κB binding to a human NF-κB DNA site (20), suggesting conservation of NF-κB function across evolution. In this study, we undertook a systematic functional comparison of human and *Nematostella* NF-κB p50 proteins. Our high-throughput protein/DNA binding analysis confirmed the data obtained by Sullivan *et al.* (20) and demonstrated overall similar DNA binding specificities of the mammalian and *Nematostella* NF-κB proteins. Nonetheless, we discovered significant differences in their interactions with cofactors. NF-κB interaction with cytoplasmic IκB molecules remained conserved during evolution, whereas its binding interface with co-regulators of the Bcl3 family has changed. This suggests that the interface between NF-κB and its IκB coactivators may be under evolutionary pressure from invading pathogens, for which blocking NF-κB-mediated immune responses would be advantageous.

## EXPERIMENTAL PROCEDURES

### 

#### 

##### Bioinformatics

Sequence alignments were performed using the ClustalW 2.0 tool (European Bioinformatics Institute).

##### Plasmids and Reagents

*nfkb1/2*^−/−^ murine embryonic fibroblasts, kindly provided by Dr. Alexander Hoffmann (University of California), and HEK293ET cells were cultured in Dulbecco's modified Eagles medium (PAA Laboratories GmbH) supplemented with 10% fetal bovine serum (Invitrogen) and 1% penicillin/streptomycin (PAA Laboratories GmbH) at 37 °C in 5% CO_2_ and 95% humidity. Mouse monoclonal antibodies against the His tag (70796-3, Novagen) and against the Myc tag (clone 9E10, Santa Cruz Biotechnology), protein G-Sepharose (GE Healthcare), and FLAG peptide and anti-FLAG-agarose (Sigma) were used. Human and *Nematostella* NF-κB p50 cDNAs was generated from human 293ET cell or *Nematostella* polyp total RNA, respectively, and subcloned into the pEAK8-Myc and pETM11-His vectors for mammalian and bacterial expression purposes. The cloned *Nematostella* p50 cDNA used in this study corresponds to the Ser-67 allele of the gene (20). NvBcl3 and NvIκB was amplified from *Nematostella* polyp cDNA and subcloned into the pBent2 vector with an N-terminal FLAG tag. Deletion and point mutants of human and *Nematostella* p50 proteins were generated by PCR using wild-type cDNA templates and subcloned into the pEAK8 vector as fusion constructs labeled with Myc or *Renilla* luciferase tags at their N termini. A 700-bp-long fragment of the *Nvbcl3* gene promoter, upstream of the first coding ATG, was amplified from *Nematostella* polyp genomic DNA and cloned into the pGL3-Basic vector (Promega) to drive expression of firefly luciferase. *Renilla* luciferase-tagged NF-κB and IκBα constructs were kindly provided by Stuart Bloor (MRC Laboratory of Molecular Biology, Cambridge, United Kingdom). All of the generated constructs were confirmed by sequencing. The pGL3-*lcn2*-luc reporter and pcDNA-FLAG-HsIκBζ expression plasmids have been described previously (21). pNF-κB-luc and pRL-TK were purchased from Clontech.

##### Electroporation, RNA Extraction, cDNA Synthesis, and Quantitative PCR (qPCR)

Murine embryonic fibroblasts (10^7^ cells/cuvette) were electroporated with 5 μg of pEAK8-Myc vector, 1 μg of pmaxGFP® (Lonza), and 4 μg of a carrier DNA (10 μg in total) using the Amaxa MEF1 Nucleofector kit (Lonza) according to the manufacturer's instructions. Three days after electroporation, cells were stimulated with 1 μg/ml LPS (Alexis Biochemicals) for 1 and 4 h or left unstimulated before collection. Total RNA was extracted from cells using the Qiagen RNeasy mini kit (Qiagen). Cells were lysed in Buffer RLT (Qiagen), and cDNA synthesis was performed using the extracted RNA, an oligo(dT) primer, and SuperScript III reverse transcriptase (Invitrogen). The cDNA was PCR-amplified using EfficienSee FAST qPCR MasterMix Plus dTTP (Eurogentec). The TaqMan gene expression assays for mouse Hprt (housekeeper control) and Lcn2 were acquired from Applied Biosystems.

##### Transfection and Reporter Assays

293ET cells were transfected in 96-well plates using Lipofectamine 2000 (Invitrogen). The pEAK8-Myc-NF-κB p50 (wild-type or mutant) and/or pBent2-FLAG (encoding IκB proteins) expression construct (10 ng/well each) was cotransfected along with a given firefly luciferase plasmid (pNF-κB-luc, pNGAL-luc, or pNvBcl3(−700)-pro-luc) and the pRL-TK plasmid (10 ng/well each). In the experiments shown in Figs. 4*B* and 5*A*, a concentration range of the IκB-encoding plasmids was used: 3 and 10 ng (Fig. 4*B*) and 2, 5, and 10 ng (Fig. 5*A*) per well. One day after transfection, cells were lysed, and luciferase activities were measured in lysates using the Dual-Glo luciferase assay kit (Promega). For stimulation experiments, cells were stimulated the next day after transfection with 10 ng/ml TNF-α (PeproTech) for 6 h before collection. Data are presented as means ± S.D. from triplicate wells of a representative experiment.

##### Protein Expression and Purification

Expression constructs for NF-κB dimers used in this study were created as described (22). Briefly, pET vectors for expression in BL21(DE3) *Escherichia coli* (Merck) were used to produce His-tagged recombinant proteins. Proteins were overexpressed through induction with 0.2 mm isopropyl β-d-thiogalactopyranoside at 30 °C for 5 h. Pellets of cells were harvested in nickel-nitrilotriacetic acid binding buffer with added EDTA-free protease inhibitor (Roche Applied Science) and pulse-sonicated for 2 min, and debris was removed by centrifugation at 16,000 × *g*. NF-κB proteins were purified by affinity chromatography in two steps: using first the nickel-nitrilotriacetic acid His-Bind resin system (Merck) and then biotinylated DNA oligonucleotides attached to streptavidin-agarose (Sigma). The bound proteins were eluted in the high-salt buffer (50 mm Tris-HCl (pH 8.0), 0.1 mm EDTA, 500 mm NaCl, 10% glycerol, and 0.01% Nonidet P-40) as described (22).

##### DNA Affinity Protein Purification

5′-Biotinylated DNA oligonucleotides containing the NF-κB site from the 3′-UTR of the human TNF promoter (5′-biotin-(AGCT)GGGCAT**GGGAATTTCC**AACTCT-3′) or the control sequence (5′-biotin-(AGCT)GGGCAT**AAACCGGGTT**AACTCT-3′) were used for NF-κB purification as described (22). Briefly, the DNA oligonucleotides were immobilized on streptavidin-agarose (Pierce). Cell pellets were lysed in protein/DNA binding buffer (50 mm Tris-HCl (pH 7.4), 50 mm NaCl, 0.1% Triton X-100, 10% glycerol, and protease inhibitors) and subjected to a French press, and the soluble fractions (separated from insoluble debris by ultracentrifugation) were incubated for 2 h with the DNA sorbent at room temperature with shaking. The proteins were eluted in high-salt buffer.

##### SDS-PAGE

The eluates were separated by SDS-PAGE using precast 4–12% denaturing gels (Invitrogen), which were Coomassie Blue-stained with the InstantBlue solution (Expedeon).

##### Protein Binding Microarrays

We designed 8 × 15K Agilent arrays using eArray as we have described in detail previously (23). Briefly, the canonical NF-κB consensus binding sequence GGRRNNYYCC was expanded into the RGGRNNHHYYB 11-mer motif, which was processed using the principal coordinate method (24). The outcome was 803 DNA sequences that are representative of the “k-mer space” encompassed by the expanded motif. The resulting *z*-score was obtained using log_2_-transformed intensities, and the median of replicates was calculated for each probe within every array (supplemental Table 1). The binding affinities for each protein were calculated using three technical replicates. The Cy3 values from the double stranding were used to normalize the Cy5 values of the protein/DNA binding. To approximate binding affinity values between proteins, these values were logged (log_2_), and a *z*-score was created using the following formula: *z*-score = (log_2_ value − median of array)/S.D. of array. The *z*-scores were used as input for the MultiExperiment Viewer software (25, 26) to create a heat map.

##### LUMIER

Luminescence-based mammalian interactome (LUMIER) mapping (27) was used to rapidly test protein/protein interactions between NF-κB and IκB proteins. Two putative interactors fused to either N-terminal FLAG or *Renilla* luciferase tags were coexpressed in 293ET cells. One day after transfection, the cells were lysed in immunoprecipitation buffer (10% glycerol, 150 mm NaCl, 0.1%. Triton X-100, 20 mm Tris-HCl (pH 7.4), 5 mm EDTA, and protease inhibitors), and the post-nuclear supernatants were incubated for 2 h with FLAG-agarose. The beads were then washed four times with immunoprecipitation buffer, and the proteins were eluted for 30 min with FLAG peptide diluted to a final concentration of 150 μg/ml in *Renilla* lysis buffer (Promega). The luciferase activity was measured in the eluates and total lysates using the *Renilla* luciferase assay system (Promega). The magnitude of luciferase activity correlates with the binding affinity within a pair of interactors. The data are labeled as -fold binding and are presented as the ratio of luciferase activity in eluates and lysates normalized against the control (empty vector).

##### ChIP

A total of 10^7^ HEK293ET cells were fixed by adding 1% formaldehyde (final concentration) for 5 min at room temperature. Nuclear extracts were subjected to 6× 12-s pulses of sonication using a Vibra-Cell VCX130 processor (Sonics) at 20% amplitude. For immunoprecipitation reaction, nuclear extracts were precleared with a protein G-Sepharose bead slurry (GE Healthcare) for 2 h and then incubated with 2 μg of 9E10 or isotype control antibodies overnight at 4 °C with rotation. Immunocomplexes were collected with protein G-Sepharose beads for 30 min, rigorously washed, and eluted. Cross-linked protein-DNA complexes were reversed by incubation overnight at 65 °C, and DNA fragments were purified using the QIAquick PCR purification kit (Qiagen). The immunoprecipitated DNA fragments were interrogated by real-time PCR using SYBR Premix Ex Taq II Master Mix (Takara Bio) and the indicated primers for the TNF promoter (5′-GGAAGCCAAGACTGAAACCAGCA and 5′-CCGGGAATTCACAGACCCCACT) and IL-10 promoter (5′-CCTGTGCCGGGAAACCTTGATTGTGGC and 5′-GTCAGGAGCACCAGGCAACAGAGCAGT) regions. Data were analyzed using Roto-Gene 6000 software (Corbett Life Science). All primer sets were tested for specificity and equal efficiency before use.

## RESULTS

### 

#### 

##### The DNA Binding Properties of NF-κB Are Evolutionarily Conserved

**FIGURE 1.**
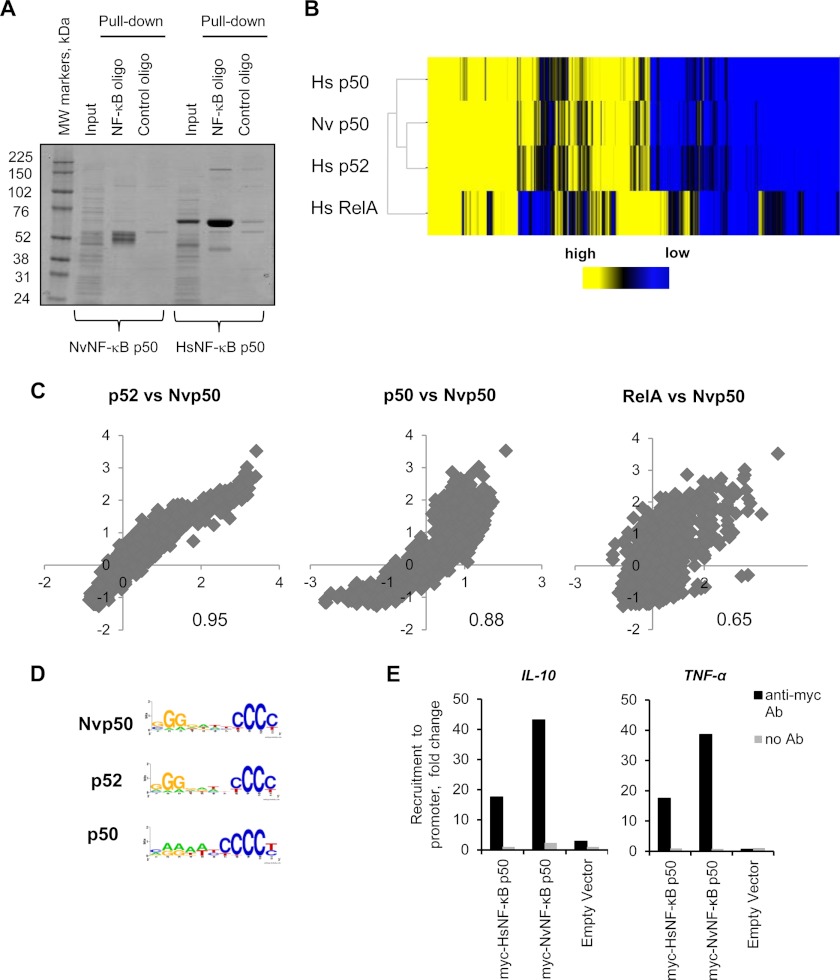
**DNA binding profiles of *Nematostella* and human NF-κB dimers.** *A*, NF-κB proteins were expressed in *E. coli*, and the soluble fractions of bacterial lysates (*Input*; separated from insoluble debris by centrifugation) were incubated for 2 h at room temperature with streptavidin-agarose attached to biotinylated DNA oligonucleotides containing NF-κB-binding (from the 3′-region of the TNF gene) or control sequences. The bound proteins were eluted with high-salt buffer, and the eluates were subjected to SDS-PAGE, followed by Coomassie Blue staining. *B*, a heat map of the binding profiles based on the microarray analysis of four NF-κB dimers (presented in *columns*) was generated using MultiExperiment Viewer (25, 26). The *z*-scores (supplemental Table 1) of each individual protein were used as input for MultiExperiment Viewer. Within the heat map, probes that contain the 803 11-mer sequences and represent k-mer space given by the consensus sequence RGGRNNHHYYB can be found as *rows*. A *color gradient* reflects the binding affinity *z*-scores of NF-κB dimers for a probe, where high-affinity probes (positive values) are shown in *yellow*, *z*-scores near zero are shown in *black*, and low-affinity probes (negative values) are shown in *blue* (see *side bar*). Hierarchical clustering was used to describe relationships between binding profiles of the different dimers (Euclidean distance correlation and complete linkage analysis). *C*, pairwise comparisons of the DNA binding profiles of *Nematostella* and human NF-κB dimers based on the array analysis. The value at the bottom of each graph is the correlation coefficient for the pair in the graph. These graphs were built for the *z*-score data sets. *z*-scores were obtained using log_2_-transformed intensities, and the median of replicates were calculated for each probe within every array. *D*, DNA barcodes of the NF-κB proteins based on the top 20 highest binding motifs for each individual protein were created using WebLogo online software. *E*, recruitment of human and *Nematostella* NF-κB p50 to human gene promoters. Plasmids encoding Myc-tagged HsNF-κB p50 and NvNF-κB p50 proteins were transfected into human 293ET cells. One day after transfection, cells were lysed, and the lysates were subjected to ChIP using anti-Myc (clone 9E10) or IgG control antibodies. The NF-κB recruitment to gene promoters was analyzed by qPCR of the precipitated DNA using specific primers to the human TNF and IL-10 gene promoters. The data are presented as -fold change over a negative IgG control.

An amino acid sequence alignment of NF-κB proteins from multiple mammalian species shows great conservation of their RHDs (8). We wished to systematically characterize the DNA binding preferences of NvNF-κB p50 and its *Homo sapiens* (Hs) orthologs NF-κB1 (HsNF-κB p50) and NF-κB2 (HsNF-κB p52). The proteins were expressed in *E. coli* and purified by DNA affinity chromatography, and their functional activity was confirmed using specific and scrambled NF-κB-binding sequences in an oligonucleotide pulldown assay (Fig. 1*A*). Used as an additional control, a DNA-binding mutant of NvNF-κB p50, FRY → AAA, failed to bind NF-κB-specific DNA (data not shown).

Next, the binding of NvNF-κB p50 to 803 11-mer sequences within the generalized NF-κB consensus sequence RGGRNNHHYYB flanked by four distinct flanking sequences was examined using double-stranded DNA microarrays essentially as described (23). We built a heat map of NvNF-κB p50 binding by arranging variant 11-mer sequences in columns and color-coding the ranked binding affinities for these sequences from high (*yellow*) to low (*blue*) (Fig. 1*B*). The NvNF-κB p50 binding profile was compared with those of HsNF-κB p50, HsNF-κB p52, and another mammalian homodimer, NF-κB RelA. As expected, HsNF-κB p50 and HsNF-κB p52 had the most similar profiles to NvNF-κB p50 (*z*-score correlation coefficients of 0.88 and 0.95, respectively), whereas the RelA binding profile was the most distant (*z*-score coefficient of 0.65) (Fig. 1*C*). The correlations between *z*-scores of the binding affinities of the selected NF-κB dimers are shown in supplemental Table 1, and they were comparable with previously determined correlations within the HsNF-κB family (23).

Despite a clear conservation of the 3′-pyrimidine-half DNA consensus sequences, *i.e.* a CC duplex at positions 9 and 10 (or on the complementary strand, a GG duplex at positions 2 and 3), we also observed differences in the DNA barcode when we compared the top 20 highest binding motifs of the individual NF-κB proteins (Fig. 1*D*). The signature GG*xxxxxx*CC symmetry of the canonical NF-κB motif observed in the case of human p52 and *Nematostella* p50 is lost in human p50, which has evolved to have a larger affinity for N_6_CCCC(T/C) sequences. The latter observation is supported by a previous study that reported the DNA binding preferences of mammalian NF-κB dimers, including p50 (28). Nevertheless, taking into account the evolutionary distance of 600 million years between *Nematostella* and mammals (29), the *in vitro* DNA binding specificities of NF-κB orthologs are remarkably similar. This is in contrast to noticeable differences between the DNA binding patterns of mammalian NF-κB paralogs (human RelA *versus* NF-κB1/2) (Fig. 1*B*).

To compare the efficiency of *in vivo* binding of HsNF-κB1 p50 and NvNF-κB p50 to the NF-κB-regulated human gene promoters, NF-κB proteins were cloned into a mammalian expression vector containing a Myc tag and expressed in the human cell line HEK293ET. The cell lysates were subjected to ChIP using anti-Myc (clone 9E10) or IgG control antibodies, followed by qPCR with specific primer sets. HsNF-κB1 p50 and NvNF-κB p50 proteins showed a similar degree of recruitment to the human TNF and IL-10 promoters (Fig. 1*E*). Therefore, both DNA binding specificity and genomic recruitment are similar between human and *Nematostella* NF-κB orthologs.

##### NF-κB Interactions with IκBα Are Evolutionarily Conserved

**FIGURE 2.**
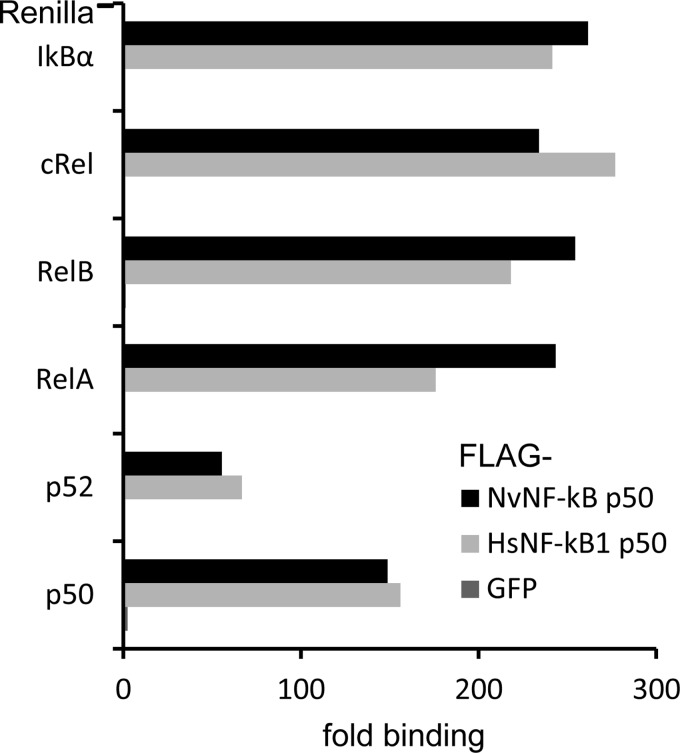
**Conservation of NF-κB protein/protein interactions.** FLAG- and *Renilla* luciferase-tagged proteins were expressed as pairs in 293ET cells. The cell lysates were subjected to immunoprecipitation using FLAG-agarose, the proteins bound to the sorbent were eluted with FLAG peptide, and the luciferase activities were measured in the eluates and total lysates. The data are shown as the ratio of luciferase activity in eluates and lysates normalized against the control (empty vector).

The NF-κB dimerization region is well conserved among various animal NF-κB orthologs (8) and includes a nuclear localization sequence (NLS) of NF-κB, which is recognized by IκB proteins (30). To examine whether NvNF-κB p50 is able to form dimers with different human NF-κB subunits and IκBα, we employed a LUMIER assay (27), which allows measurement of potential binding between a pair of proteins. NvNF-κB and HsNF-κB were tagged with FLAG, whereas all of the human NF-κB subunits and IκBα carried a *Renilla* luciferase tag. The individual pairs of proteins were expressed in HEK293ET cells and immunoprecipitated from cell lysates using FLAG-agarose. Both NvNF-κB and HsNF-κB interacted strongly with IκBα and all Rel proteins, whereas a negative control protein, FLAG-GFP, did not (Fig. 2). Thus, the dimerization properties of NF-κB and its interaction with cytoplasmic IκBs are evolutionarily conserved.

##### HsIκBζ Discriminates between Human and Nematostella NF-κB

**FIGURE 3.**
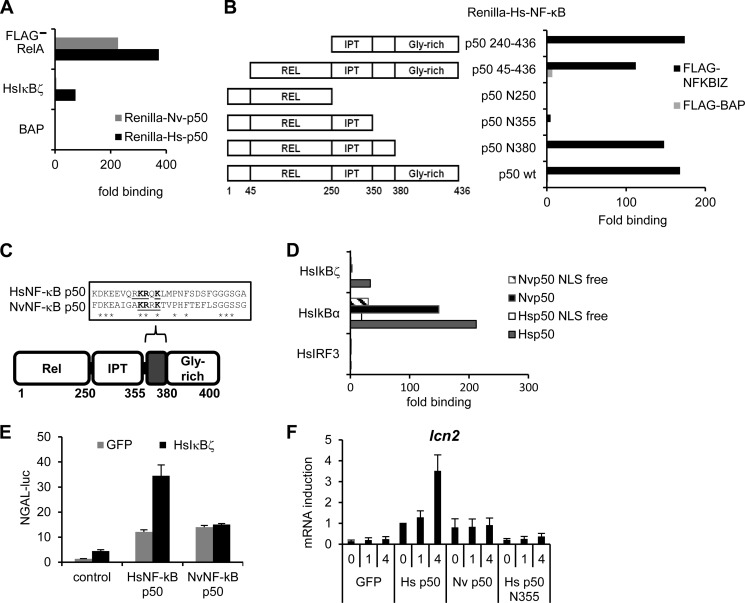
**Human IκBζ discriminates between human and *Nematostella* NF-κB.** *A*, *Renilla* luciferase-tagged NF-κB p50 proteins coexpressed with either FLAG-tagged RelA or HsIκBζ. The protein complexes were pulled out from cell lysates using FLAG-agarose, the bound proteins were eluted with FLAG peptide, and the luciferase activities in the lysates and eluates were measured. The data are shown as -fold binding compared with the control (bacterial alkaline phosphatase). *B*, the deletions mutants of human NF-κB p50, N-terminally tagged with *Renilla* luciferase (*left*), were coexpressed with either bacterial alkaline phosphatase (control) or FLAG-tagged HsIκBζ. The protein/protein interactions were analyzed as described for *A. Rel*, RHD. *C*, the HsIκBζ-binding region of HsNF-κB p50 was aligned with the corresponding region of NvNF-κB p50 using ClustalW software. The NLS motif is *underlined. D*, LUMIER assay of selected NF-κB/IκB interactions. *Renilla* luciferase-tagged wild-type human and *Nematostella* NF-κB p50 or NLS mutant versions were coexpressed with either IRF3 (control) or FLAG-tagged IκB proteins in 293ET cells. The protein complexes were pulled out from cell lysates using FLAG-agarose, the bound proteins were eluted with FLAG peptide, and the luciferase activities in the lysates and eluates were measured. The data are shown as -fold binding compared with the control (IRF3). *E*, HsNF-κB p50 or NvNF-κB p50 was coexpressed with or without HsIκBζ and the *NGAL*/*lcn2* promoter-based luciferase reporter. The luciferase activities were measured in cell lysates. The data are shown as -fold induction over the control (empty vector) as means ± S.D. of triplicate experiments. *F*, plasmids expressing either GFP or NF-κB proteins were delivered into *nfkb1/2*^−/−^ murine embryonic fibroblasts using electroporation. Two days later, cells were stimulated with 1 μg/ml LPS for 1 and 4 h and subjected to RNA extraction. Induction of *lcn2* mRNA synthesis was measured by qPCR. The data are shown as means ± S.D. of a representative experiment.

The mammalian NF-κB p50 subunit lacks a transactivation domain and requires a cofactor like HsIκBζ to drive transcription of its dedicated genes (17). We used the LUMIER assay to compare HsIκBζ binding of HsNF-κB p50 and NvNF-κB p50. FLAG-tagged HsIκBζ was expressed along with the *Renilla* luciferase-tagged NF-κB proteins. FLAG-RelA was used as a confirmed binding partner of p50. Interestingly, HsNF-κB, but not NvNF-κB, bound to HsIκBζ, whereas both proteins bound well to RelA (Fig. 3*A*).

It is not entirely known how NF-κB interacts with HsIκBζ, with the C terminus of p50 being proposed to play role (31). To clarify which region of HsNF-κB interacts with HsIκBζ, we constructed several deletion mutants of HsNF-κB p50 and tested them in the LUMIER assay for their affinity to HsIκBζ (Fig. 3*B*). The DNA-binding domain of HsNF-κB p50 was dispensable for its interactions with HsIκBζ, but so was its C terminus, as HsNF-κB mutant N380 (amino acids 1–380) could still bind to HsIκBζ (Fig. 3*B*). However, HsNF-κB p50 mutant N355 (amino acids 1–355) failed to interact with HsNF-κB (Fig. 3*B*). The alignment of the region encompassing amino acids 355–380 of human and *Nematostella* NF-κB p50 demonstrated that it is only 40% identical between the species and encompasses the conserved NLS motif (amino acids KR*x*K, *underlined* in Fig. 3*C*). To further dissect the contribution of the NF-κB p50 evolving *versus* conserved amino acids in recognition of HsIκBζ, we used NLS-free mutants of HsNF-κB p50 and NvNF-κB p50 in the LUMIER binding assay and found that the NLS is important for NF-κB binding to HsIκBζ, as well as to HsIκBα, which was used as a control (Fig. 3*D*). Therefore, we concluded that the sequences outside of the NLS are responsible for differential recognition of HsIκBζ by human and *Nematostella* NF-κB p50.

Next, we sought to examine the functional consequences of the differential HsIκBζ/p50 binding. We coexpressed HsIκBζ with human or *Nematostella* NF-κB p50 and a luciferase reporter driving the expression of the HsIκBζ-dependent gene *NGAL*/*lcn2* in human 293ET cells (Fig. 3*E*). HsNF-κB p50 co-induced greater reporter activation compared with NvNF-κB p50. Finally, we used a genetic complementation test in *nfkb1/2*^−/−^ murine embryonic fibroblasts (32) to examine expression of the endogenous *lcn2* gene. Introduction of human p50, but not its *Nematostella* counterpart or the human p50 mutant lacking the C-terminal HsIκBζ-binding region, could restore LPS-inducible expression of *lcn2* in these cells (Fig. 3*F*).

In summary, these data indicate that HsIκBζ can discriminate between human and *Nematostella* NF-κB proteins. Thus, we hypothesized that the IκB/p50 interaction interface might have undergone changes during evolution.

##### NvBcl3 Discriminates between Human and Nematostella NF-κB

**FIGURE 4.**
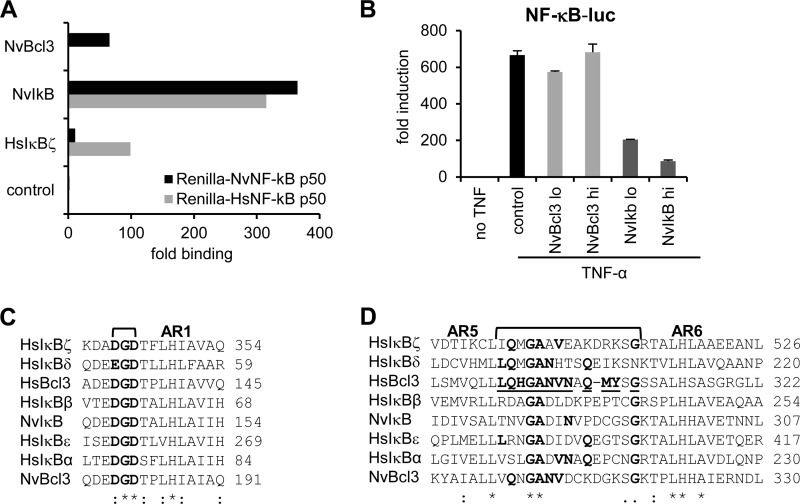
**Distinct properties of *Nematostella* IκB and Bcl3 proteins.** *A*, LUMIER assay of selected NF-κB/IκB interactions. The *Renilla* luciferase-tagged human and *Nematostella* NF-κB p50 proteins were coexpressed with one of the FLAG-tagged IκB proteins in 293ET cells. The protein complexes were pulled out from cell lysates using FLAG-agarose, the bound proteins were eluted with FLAG peptide, and the luciferase activities in the lysates and eluates were measured. The data are shown as -fold binding compared with the control (bacterial alkaline phosphatase). *B*, 293ET cells seeded in 96-well plates were transfected with 3 or 10 ng (*lo* and *hi*, respectively)/well NvBcl3- or NvIκB-encoding plasmid and 10 ng/well pNF-κB-luc reporter plasmid. One day later, the cells were stimulated for 6 h with 10 ng/ml TNF-α before collection. The luciferase activities were measured in cell lysates. The data are shown as -fold induction over the control (empty vector) as means ± S.D. of a triplicate experiment. *C* and *D*, the amino acid sequence alignments of human and *Nematostella* IκB proteins. Sequences were aligned using ClustalW software, and the ankyrin repeats (*AR*) were labeled according to a previously used annotation (30). Conserved residues are shown in *boldface*, and the predicted loop residues of HsBcl3 contacting HsNF-κB p50 are *underlined*.

To test our hypothesis, we first examined binding of the *Nematostella* homolog of HsIκBζ to both HsNF-κB p50 and NvNF-κB p50. Two IκB-related proteins, NvIκB and NvBcl3, have so far been identified in *Nematostella* (8, 19). NvBcl3 is phylogenetically related to mammalian Bcl3 and HsIκBζ and appears to be localized in both the cytosol and nucleus (8, 19). NvIκB is similar to mammalian cytoplasmic IκBs: it has an N-terminal IκB kinase phosphorylation motif, and it was shown to sequester NvNF-κB in the cytoplasm when ectopically expressed in mammalian cells (8, 19). We tested the binding of NvIκB and NvBcl3 to HsNF-κB p50 and NvNF-κB p50 by LUMIER. NvIκB strongly bound to both human and *Nematostella* NF-κB p50, whereas NvBcl3 bound only to NvNF-κB (Fig. 4*A*). Moreover, the expression of NvIκB, but not NvBcl3, inhibited the TNF-α-induced pNF-κB-luc reporter in human HEK293ET cells (Fig. 4*B*), confirming the fact that NvIκB, but not NvBcl3, can bind human NF-κB.

To better understand the molecular basis of the differential recognition of NF-κB by cytosolic and nuclear IκB proteins, we considered crystallographic and molecular modeling studies of complexes between IκB and NF-κB (30). First, they showed that the IκBα DGD motif interacts with the NF-κB p50 NLS (30). We performed an amino acid sequence alignment of the DGD region for all human and *Nematostella* IκB proteins and found that it is conserved in IκB and Bcl3-like proteins (Fig. 4*C*). This supports our finding that the NLS is crucial for NF-κB binding by both groups of IκBs (Fig. 3*D*). Second, they predicted that loops located between ankyrin repeats 4, 5, 6, and 7 of IκBs are involved in NF-κB binding. For example, the loop located between ankyrin repeats 5 and 6 of human Bcl3 contains several residues contacting the NF-κB p50 dimer (30). We generated an amino acid sequence alignment of this region for all human and *Nematostella* IκB proteins and found that there is only a little conservation in the predicted residues contacting NF-κB (Fig. 4*D*). It is therefore possible that the dynamic evolution of the indicated IκB loops is responsible for the differential recognition of NF-κB by cytosolic and nuclear IκB proteins.

##### Inhibition of NF-κB Activity by Cnidarian IκB Proteins

**FIGURE 5.**
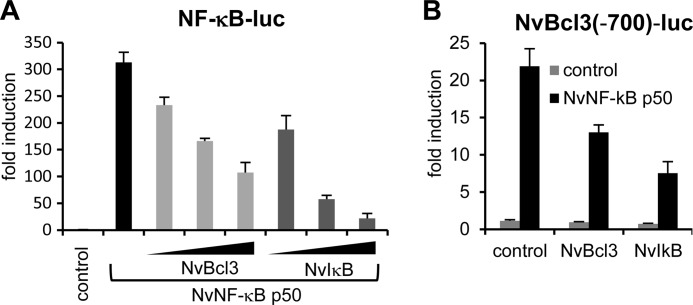
***Nematostella* NF-κB activity is suppressed by *Nematostella* IκB proteins.** *A* and *B*, 293ET cells seeded in 96-well plates were transfected for 24 h with either 10 ng/well pNF-κB-luc (*A*) or 10 ng of *Nvbcl3* gene promoter-based (*B*) luciferase reporter plasmid and increasing amounts of the NvBcl3- or NvIκB-expressing constructs (2, 5, and 10 ng/well in *A* and 10 ng/well in *B*) or control plasmid (empty vector). One day later, the cells were lysed, and the luciferase activities were measured. The data are shown as -fold induction over the control (empty vector) as means ± S.D. of a triplicate experiment.

NvIκB and NvBcl3 have been shown previously to inhibit Nvp50-mediated activity of the synthetic pNF-κB-luc reporter (19). We extended these observations (Fig. 5*A*) to NF-κB-dependent *Nematostella* gene expression by analyzing the activity of a reporter construct driven by a 700-nucleotide upstream region of the predicted NvNF-κB target gene, *Nvbcl3* (Fig. 5*B*). NvNF-κB induced pNvBcl3(−700)-luc reporter activity, whereas NvIκB or NvBcl3 attenuated this induction (Fig. 5*B*). These data suggest that inhibition of NF-κB activity by IκB proteins is not a recent property but that it originated before the separation of cnidarian and triploblastic animal lineages.

## DISCUSSION

NF-κB is a key regulator of gene expression in inflammation and other immune responses, but little is known about the evolution of the NF-κB system. In this study, we compared the functional properties of human and *Nematostella* NF-κB proteins and found that their DNA binding specificities *in vitro* and *in vivo* are largely conserved and that evolutionarily conserved immune genes have been regulated by NF-κB since, at least, the origin of cnidarians. We have shown that both human and *Nematostella* NF-κB can interact equally well with all mammalian NF-κB subunits and inhibitors HsIκBα and NvIκB. However, the binding of NF-κB to the Bcl3 family of proteins is not conserved: HsIκBζ and NvBcl3 can discriminate between human and *Nematostella* NF-κB and bind only the transcription factor from their own species.

The similarity in the functional properties of NF-κB proteins reported in this study is a direct result of evolutionary conservation of the amino acid sequence of NF-κB (8). For example, the RFRYXCE region of the RHD, responsible for p50 binding to DNA (33, 34), is highly conserved between *Nematostella* and mammalian NF-κB proteins (8). Still, paralog formation in the vertebrate NF-κB lineage appears to have led to a specialization of DNA recognition among different NF-κB subunits within the limits imposed by structural constraints of the conserved elements of the RHD fold. Although human p50 and p52 subunits are most closely related to *Nematostella* p50 and have similar DNA binding specificities, a subunit more distantly related to them, NF-κB RelA, shows divergent DNA sequence preferences. Thus, the heterodimerization of NF-κB paralogs along with the dynamic evolution of the NF-κB/DNA binding barcode might have stimulated complexity and flexibility in the NF-κB transcriptome in vertebrates.

NF-κB has been shown to control immune gene expression in both *Drosophila* and mammals (9, 35). With the overall conservation of NF-κB/DNA binding across the animal kingdom, it is logical to speculate that *Nematostella* NF-κB may regulate immune genes in its own host also. A previous study predicted potential gene targets of NF-κB in *Nematostella* and showed that NvIκB gene promoter activity can be induced by the ectopic coexpression of NvNF-κB in human cells (19). Here, we cloned the promoter region of another IκB gene, *Nvbcl3*, whose mammalian paralog expression is known to be NF-κB-dependent (18). As expected, *Nematostella* NF-κB triggered the *Nvbcl3* promoter-driven luciferase expression. This indicates that evolutionarily conserved immune genes, such as IκBs, have been regulated by NF-κB since, at least, the origin of cnidarians.

It is significant that the entire amino acid sequence of NF-κB is not conserved. The 355–380-amino acid region next to the IPT dimerization domain shows only a partial similarity between human and *Nematostella* p50 proteins. This region is known to be a binding hub for IκB proteins (30). Here, we have demonstrated that it is recognized differentially by the two subfamilies of IκBs. Both human and *Nematostella* IκBα proteins bind to human and *Nematostella* NF-κB equally well and inhibit the transcriptional activity of both proteins. In contrast, NvBcl3 can bind only to *Nematostella* NF-κB, but not human NF-κB, whereas HsIκBζ fails to recognize the cnidarian NF-κB protein. The selectivity of the Bcl3 family proteins in NF-κB binding is reflected in their function. In association with human NF-κB, HsIκBζ has been shown to drive *lcn2* gene transcription (36). We have demonstrated that ectopic HsIκBζ triggers less *lcn2* reporter activity or *lcn2* gene expression when coexpressed with *Nematostella* NF-κB than when it is paired with human NF-κB. Conversely, NvBcl3 can inhibit only NvNF-κB-induced reporter activation but fails to suppress TNF-α-induced NF-κB activation in human cells.

What is the molecular basis of the differential recognition of NF-κB by the two IκB subfamilies? The *in silico* modeling suggests that loops of the ARDs, which show great sequence diversity, confer the specificity of NF-κB/IκB interactions (30). However, these predictions alone do not explain why cytoplasmic IκBs bind strongly to both human and *Nematostella* NF-κB proteins, whereas the Bcl3-type IκBs are more selective and weaker binders. We hypothesize that the cytoplasmic IκBs bind to NF-κB proteins via conserved elements, whereas the Bcl3-type IκBs bind via the variable ARD loops, but more work is needed to address this issue experimentally.

The interface between NF-κB and its IκB family coactivators may be under evolutionary pressure from invading pathogens keen to hamper NF-κB-mediated immune responses. Interestingly, a recent study has shown that a measles virus encodes an IκB-like protein, which blocks the NF-κB response by retaining RelA in the cytoplasm (37). Another example is an insect virus encoding eight proteins orthologous to *Drosophila* IκB called Cactus, which have been shown to block the midgut melanotic response during infection (38). Therefore, pathogen-derived IκB molecules can compete with NF-κB coactivators, *i.e.* the Bcl3-type proteins, to suppress the host immunity. On the other hand, the host IκB inhibitors do not interfere with a pathogen's agenda, and therefore, there is less evolutionary pressure on them.

In summary, in addition to demonstrating a remarkable conservation of the NF-κB/DNA binding, dimerization properties, and interactions with cytosolic IκBs, we have uncovered a previously unknown phenomenon of the evolving interaction interface between NF-κB and nuclear IκBs. It is conceivable that a stronger evolutionary pressure on the NF-κB interaction with coactivator nuclear IκB molecules has been exerted by the pathogens trying to evade the host immunity.

## Supplementary Material

Supplemental Data

## Footnotes

4 The abbreviations used are:

Nv: *N. vectensis*
RHD: Rel homology domain
ARD: ankyrin repeat domain
qPCR: quantitative PCR
Hs: *H. sapiens*
NLS: nuclear localization sequence.
