# Supplementary material for: Cross-species Analysis Reveals Evolving and Conserved Features of the Nuclear Factor κB (NF-κB) Proteins
Source: J Biol Chem. 2013 Mar 18;288(16):11546–54. doi: 10.1074/jbc.M113.451153 (PMC3630861; doi:10.1074/jbc.M113.451153)
Supplement: Supplemental Data [file supp_288_16_11546__index.html]

Cross-species Analysis Reveals Evolving And Conserved Features of the NF-κB Proteins — Cross-species Analysis Reveals Evolving and Conserved Features of the Nuclear Factor κB (NF-κB) Proteins — Evolving and Conserved Features of the NF-κB Proteins — Supplemental Data 

# Cross-species Analysis Reveals Evolving and Conserved Features of the Nuclear Factor κB (NF-κB) Proteins

## Supplemental Data

**Files in this Data Supplement:**

- Supplemental Table 1 (.xls, 245 KB) - Supplemental Table 1. Array Datasets
